# Sulfasalazine, an inhibitor of the cystine-glutamate antiporter, reduces DNA damage repair and enhances radiosensitivity in murine B16F10 melanoma

**DOI:** 10.1371/journal.pone.0195151

**Published:** 2018-04-12

**Authors:** Masaki Nagane, Eiichi Kanai, Yuki Shibata, Takuto Shimizu, Chie Yoshioka, Takuya Maruo, Tadashi Yamashita

**Affiliations:** 1 Laboratory of Biochemistry, School of Veterinary Medicine, Azabu University, Sagamihara, Kanagawa, Japan; 2 Laboratory of Veterinary Radiology, School of Veterinary Medicine, Azabu University, Sagamihara, Kanagawa, Japan; 3 Teaching Animal Hospital, Azabu University, Sagamihara, Kanagawa, Japan; University of South Alabama Mitchell Cancer Institute, UNITED STATES

## Abstract

The sodium-independent cystine-glutamate antiporter plays an important role in extracellular cystine uptake. It comprises the transmembrane protein, xCT and its chaperone, CD98. Because glutathione is only weakly cell membrane permeable, cellular uptake of its precursor, cystine, is known to be a key step in glutathione synthesis. Moreover, it has been reported that xCT expression affects the progression of tumors and their resistance to therapy. Sulfasalazine is an inhibitor of xCT that is known to increase cellular oxidative stress, giving it anti-tumor potential. Here, we describe a radio-sensitizing effect of sulfasalazine using a B16F10 melanoma model. Sulfasalazine decreased glutathione concentrations and resistance to H_2_O_2_ in B16F10 melanoma cells, but not in mouse embryonic fibroblasts. It synergistically enhanced the cyto-killing effect of X-irradiation in B16F10 cells. It inhibited cellular DNA damage repair and prolonged cell cycle arrest after X-irradiation. Furthermore, in an *in vivo* transplanted melanoma model, sulfasalazine decreased intratumoral glutathione content, leading to enhanced susceptibility to radiation therapy. These results suggest the possibility of using SAS to augment the treatment of radio-resistant cancers.

## Introduction

Intracellular glutathione (GSH), which can scavenge reactive oxygen species (ROS), contributes to the maintenance of cellular redox potential while preventing and repairing oxidative damage. GSH, a cysteine-glutamate-glycine tripeptide, and the thiol-group of cysteine can transfer a proton to reduced molecules. Because GSH is only weakly cell membrane permeable, its biosynthesis is controlled by the intracellular cysteine concentration. Therefore, inhibitors of GSH biosynthesis can act as radio-sensitizers. Several reagents that directly interfere with GSH synthase have been evaluated in previous studies. However, they showed high toxicity in clinical trials and poor specificity for tumor-associated GSH [1, 2]. An alternative strategy is needed to interfere with GSH biosynthesis for optimizing tumor radiotherapy.

The sodium-independent cystine-glutamate antiporter plays an important role in the uptake of extracellular cystine [3, 4]. This transporter comprises the transmembrane protein, xCT (also known as SLC7A11) and its chaperone, CD98. Cellular uptake of cystine, a precursor of GSH, is the key step in GSH synthesis. Moreover, it has been reported that xCT expression is associated with tumor malignancy and resistance to therapy [4, 5]. Recently, an exploratory study for xCT inhibitors showed that the non-steroidal anti-inflammatory drug, sulfasalazine (SAS) potentially inhibits xCT function [6], and Ishimoto *et al*. demonstrated the anticancer effect of SAS [7]. Sleire *et al*. have also demonstrated that SAS enhances the effect of gamma knife radiosurgery on glioma cells [8]. In addition, Rodman *et al*, have reported that GSH depletion affected the radio-/chemo-resistance of breast cancer stem cells, indicating that GSH depletion by SAS potentially sensitizes cancer cells to radiation [9]. However, the precise mechanism of the radio-sensitizing effect and tumor selectivity of SAS remains unclear. Therefore, in the present study, we clarified the effect of SAS on the radiotherapy of B16F10 melanoma *in vitro* and *in vivo*.

## Materials and methods

### Reagents

Roswell Park Memorial Institute-1640 medium (RPMI-1640; Cat. No. 043–30085), Dulbecco’s modified Eagle’s medium (DMEM; Cat. No. 189–02025), and non-essential amino acids (NEAA; Cat. No. 139–15651) were purchased from Wako Pure Chemical Industries (Osaka, Japan). SAS (Cat No. S0883) and 2’,7’-dichlorofluorescin diacetate (DCFDA; Cat. No. D6883), siRNA (Product no. Mm_Slc7a11_1916), and MISSION siRNA Universal Negative Control #2 (Product no. MISSSION_SIC-002) were purchased from Sigma-Aldrich (St. Louis, MO). Lipofectamine^®^ RNAiMAX and Opti-mem were purchased from Thermo Fisher Scientific (Waltham, MA). Water soluble tetrazolium salt-1 (WST-1; Cat. No. W201) and 1-methoxy phenazine methosulfate (Cat. No. M003) were purchased from Dojindo (Kumamoto, Japan). The following antibodies were used for western blotting and immunostaining: anti-γ-tubulin (Cat. No. ab179503, Abcam, Cambridge, United Kingdom), anti-pSer139-histone H3 (γH2AX; Cat. No. #9718, Cell Signaling Technology, Beverly, MA), Alexa Fluor 488- or 598-conjugated secondary antibodies (Thermo Fisher Scientific), anti-xCT (Cat. No. sc-98552), anti-actin (Cat. No. sc-1615), and horseradish peroxidase (HRP)-conjugated secondary antibodies (Santa Cruz Biotechnology, Santa Cruz, CA). Immobilon western HRP substrate was purchased from Merck Millipore (Billerica, MA).

### Cell culture

Murine melanoma B16F10 cells (Cat No. TKG0348, Cell Resource Center for Biomedical Research, Tohoku University, Sendai, Japan) were maintained in RPMI-1640 medium supplemented with 10% fetal bovine serum at 37°C, 5% CO_2_. Mouse embryonic fibroblasts (MEF) were maintained in DMEM supplemented with 10% fetal bovine serum and NEAA at 37°C, 5% CO_2_. MEF were used between passages 3 and 8.

### Intracellular ROS detection

Intracellular oxidative stress was evaluated by DCFDA staining, as reported [10]. Cells were treated with 0–1,000 μM SAS for 24 h. After SAS treatment, cells were stained with 20 μM DCFDA for 30 min at 37°C. Cells were trypsinized and their fluorescence intensity was analyzed using an EC800 Analyzer (Sony Biotechnology, Tokyo, Japan).

### Cell viability assay

To determine the effect of SAS and exogenous oxidative stress on cellular viability, a WST-1 viability assay was employed, as described previously [11]. Cells (1,000–2,000 per well) were seeded into all wells of 96-well plates, and medium was replaced with SAS-containing medium (0–1000 μM) for 24 h. To determine the effect of SAS on H_2_O_2_ toxicity, cells were treated with H_2_O_2_ (10–200 μM, 24 h) after SAS treatment (200 μM, 24 h). After treatment with SAS alone or H_2_O_2_ plus SAS, cells were treated with WST-1 solution (3.6 μg/μL WST-1, 70 ng/μL 1-methoxy phenazine methosulfate in 20 mM 4-(2-hydroxyethyl)-1-piperazineethanesufonic acid—KOH [pH 7.4]; Dojindo). The cells were incubated for 1 h at 37°C, and the absorbance of each well was recorded at 440 nm using a Multiskan FC microplate reader (Thermo Fisher Scientific).

### Reverse transcription–quantitative polymerase chain reaction analysis

Reverse transcription–quantitative polymerase chain reaction (RT-qPCR) analysis was performed as previously described [12]. Total RNA from B16F10 and MEF cells was isolated using TRIzol Reagent (Cat. No. 15596026, Thermo Fisher Scientific) according to the manufacturer’s instructions. Five-hundred nanograms of RNA were reverse-transcribed using ReverTra Ace qPCR RT Master Mix with gDNA remover (Cat. No. FSQ-301, TOYOBO, Osaka, Japan). Real-time PCR analysis was performed using a LightCycler Nano System together with a KAPA SYBR FAST qPCR Kit (Cat. No. KK4602, KAPA Biosystems, MA, USA). The primer sequences for qPCR were as follows: for *xCT*, 5′- ACATTCTGGAGGTCTTTGGT -3′ and 5′- GCAAGTTCAGGAATTTCACA -3′; for *β2-microglobulin* (*B2M*), 5′- TTTCTGGTGCTTGTCTCACT -3′ and 5′- GTTCAGTATGTTCGGCTTCC -3′. PCR conditions were as follows: 95°C for 3 min, followed by 45 cycles of 95°C for 10 s, 60°C for 10 s, and 72°C for 15 s. The fluorescence intensity was measured at the end of every extension phase. Relative mRNA expression was normalized to that of *B2M*, as an internal control.

### Sodium dodecyl sulfate–polyacrylamide gel electrophoresis and western blotting

Western blotting was performed as previously described, with some modifications [13]. For B16F10 and MEF, cells were collected and lysed in modified RIPA buffer (50 mM Tris-HCl [pH 7.4], 150 mM NaCl, 1 mM ethylenediaminetetraacetic acid, 1% NP-40, 0.1% sodium dodecyl sulfate (SDS), 0.1% sodium deoxycholate, and protease inhibitor cocktail) following sonication using a Bioruptor UCD-200 (BM Equipment, Tokyo, Japan) at low intensity for 3 min. For tumor and skin tissues, tissues were lysed in modified RIPA buffer using an bead beater-type homogenizer (μT-12, Taitec corporation, Saitama, Japan). Lysed cell and tissue were centrifuged at 15,000×g for 15 min at 4°C, and the supernatants were collected as protein samples. Three-fold concentrated Laemmli’s sample buffer (0.1875 M Tris-HCl [pH 6.8], 15% β-mercaptoethanol, 6% SDS, 30% glycerol and 0.006% bromophenol blue) was added to the supernatant, and samples were boiled for 3 min. Proteins were separated by SDS–polyacrylamide gel electrophoresis and transferred onto a nitrocellulose membrane (BioTrace NT; Pall Corporation, FL, USA). Transfer conditions were 100 V in Towbin buffer (25 mM Tris, 192 mM glycine, 1%SDS, and 20% methanol) for 90 min at 4°C. The membrane was probed with specific antibodies diluted with TBST (10 mM Tris-HCl [pH 7.4], 0.1 M NaCl and 0.1% Tween-20) containing 5% bovine serum albumin overnight at 4°C. After probing with HRP-conjugated secondary antibodies, bound antibodies were detected with Immobilon western HRP substrate. Densitometry was performed using ImageJ software (NIH, Bethesda, MD).

### GSH measurement

Total GSH concentration was evaluated using a Total GSH Quantification Kit (Cat No. T419; Dojindo), following the manufacturer’s instructions. Harvested cells and tumor tissue were lysed in 10 mM HCl and 1% 5-sulfosalicylic acid dihydrate (Wako Pure Chemical Industries). Lysate was centrifuged (8,000×g) and the supernatant collected. An equal volume of H_2_O was added to the supernatant and incubated with coloring reagents.

### RNA interference

Mouse xCT siRNA (Product no. Mm_Slc7a11_1916) and MISSION siRNA Universal Negative Control (Product no. MISSSION_SIC-002) were purchased from Sigma-Aldrich. Cells were reverse-transfected with these siRNAs (30 nM) with Lipofectamine^®^ RNAiMAX (4.4 μL per 35 mm dish). RNAi complex were formed in 200 μL of Opti-mem^®^ (Product no. 31985–070) for 20 min of incubation, then 2 mL of cell suspension (5×10^5^ cells per 35 mm dish) were merged into RNAi complex. At 48–72 h after transfection, cells were proceeded to further analysis.

### Clonogenic survival assay and irradiation

A clonogenic survival assay for determination of the radio-sensitizing effect of SAS was performed as previously described [14]. B16F10 cells were trypsinized, diluted, counted, and seeded into 60-mm dishes at densities of 100–10,000 cells/dish before being allowed to adhere in a 37°C incubator for 6 hours. Prior to irradiation, cells were incubated with SAS for 24 h. X-irradiation was performed with a linear accelerator (Primus MidEnergy, Siemens, Berlin, Germany). The dose rate was 2 Gy/min, determined using Fricke’s chemical dosimeter. The irradiated cells were then allowed to grow in a humidified 5% CO_2_ atmosphere at 37°C for 5 days before being fixed with methanol and stained with 0.05% crystal violet solution (Wako Pure Chemical Industries). Colonies that containing more than 50 cells were scored as surviving cells. The survival curves were then fitted to a linear–quadratic model using Origin Pro 7 data analysis software (Origin Lab Co., MA).

### Analysis of mitotic catastrophe

Radiation-induced mitotic catastrophe (MC) was analyzed as previously described, with some modifications [15]. Cells were seeded onto glass coverslips coated with poly-L-lysine (Sigma-Aldrich) and cultured overnight followed by SAS treatment (200 μM, 24 h). After 8 Gy of X-irradiation, the cells were fixed with 4% paraformaldehyde/phosphate-buffered saline (PBS) for 10 min at 4°C. After permeabilization with 0.1% Triton X-100/PBS for 5 min at 4°C, the cells were treated with blocking buffer (PBS containing 1% bovine serum albumin and 5% goat serum) for 30 min at room temperature. For MC analysis, cells were then incubated with anti-γ-tubulin antibody (1:500 in blocking buffer) at 4°C overnight, followed by Alexa Fluor^®^ 488-conjugated secondary antibody (1:2000 in blocking buffer) for 1 h. After being washed twice with PBS, the cells were incubated with 300 nM 4′,6-diamidino-2-phenylindole for 5 min at room temperature, and the coverslips were mounted with ProLong Gold antifade reagent (Thermo Fisher Scientific). At least 100 cells were counted, and the cells either with features of aberrant mitotic nuclei (micronuclei, multilobular nuclei, or fragmented nuclei) or with more than two γ-tubulin foci were scored as cells undergoing MC.

### DNA damage repair assay

DNA damage repair assays were performed as reported previously [16]. The cells were fixed and permeabilized as described in *2*.*9*. *Analysis of mitotic catastrophe*, above. The cells were then incubated with anti-γH2AX antibody (1:500 in blocking buffer) at 4°C overnight followed by incubation with Alexa Fluor^®^ 488-conjugated secondary antibody (1:2000 in blocking buffer, 1 h, room temperature). After two washes with PBS, they were incubated with 300 nM 4′,6-diamidino-2-phenylindole for 20 min at room temperature, and the coverslips were mounted with ProLong Gold antifade reagent (Thermo Fisher Scientific). Fluorescence microscopic analysis was performed using an Olympus BX61. Foci of γH2AX were manually counted in at least 100 cells.

### Alkaline comet assay

To detect primary cytotoxicity of X-irradiation, DNA strand breaks were quantified by alkaline comet assay [17]. Cells were incubated with SAS (200 μM, 24h) prior to 1 Gy of X-irradiation. After irradiation, cells were suspended in 1% low-melting point agarose (Cat No. 317–01182; Nippon Gene, Tokyo, Japan) dissolved in serum free medium. Cell suspension containing 5×10^3^ cells were applied to microscope slide that pre-coated by 1% agarose (Cat No. 313–90231; Nippon Gene, Tokyo, Japan). Slides were incubated with comet lysis buffer (1.2 M NaCl, 100 mM EDTA, 0.1% sodium lauryl sarcosinate, 0.26 M NaOH) at 4°C for overnight. Then, slides were washed twice by alkaline buffer (0.03 M NaOH, 2 mM EDTA). Genomic DNA were separated in alkaline buffer at 9 V for 15min. After being washed with distilled water, DNA were stained by Propidium iodide (10 μg/mL, 4 °C, 30min). Fluorescent images were acquired using an BZ-X700 microscope (Keyence, Osaka, Japan), and comet tail were analyzed using Image J software with OpenComet plug-in at least 30 cells per sample [18].

### Cell cycle analysis

To analyze cell cycle perturbation, DNA was stained with propidium iodide and the cells were analyzed by flow cytometry [19]. Cells were incubated with SAS prior to 1 Gy of X-irradiation. At the indicated time points, 0.5×10^6^ cells were resuspended in PBS and fixed in ice cold 70% ethanol for at least 6 h. Fixed cells were washed in PBS twice, and incubated with propidium iodide staining solution (50 μg/mL propidium iodide, 5 μg/mL RNase I, 137 mM NaCl, 2.7 mM KCl, 8.1 mM Na_2_HPO_4_, and 1.47 mM KH_2_PO_4_) at 37°C for 30 min. The DNA content of at least 10,000 cells/sample was analyzed using an EC800 Analyzer (Sony Biotechnology).

### General animal methods

All animal experiments were performed according to the established guidelines of the ‘‘Law for the Care and Welfare of Animals in Japan”, and approved by the Animal Experiment Committee of Azabu University (Approved No. 160402). Mice were housed in plastic cages in an air-conditioned room at 24 °C in a 12 h light-dark cycle (light on at 7:00 am) with food and water available ad libitum on SPF condition. Tumor-bearing mice was ethically sacrificed when the tumor volume reached 1,500 mm^3^ (or ~1.5 cm in any dimension) or a tumor burden greater than 10% of the body weight; tumor that ulcerate, become necrotic or infected, when a tumor impedes an animal’s ability to move their limbs and assume normal body postures, and any animal found to be moribund, cachectic, or unable to obtain food or water. At the end of experiments, animals will be anesthetized with 2% isoflurane and sacrificed by cervical dislocation. Death will be determined by confirming that dislocation took place if the mice head hangs loosely from the body.

### Tumor transplantation

Female C57BL/6N mice aged 7–8 weeks were purchased from Charles river laboratories Japan (Kanagawa, Japan) and maintained and inbred in the animal facility of Azabu University. For allograft transplantation, skin will be disinfected with 70% alcohol, and one million B16F10 cells were inoculated subcutaneously into the hind-legs of the mice under anesthesia by 2% of isoflurane (Intervet, Tokyo, Japan). Tumor volume was calculated as V (mm^3^) = π/6 × a × b × c, where a, b, and c are the orthogonal dimensions of the tumor. In this allograft model, we did not observe the decrease of body weight (S1 Fig).

### Immunohistochemistry

Immunohistochemistry was performed as previously described [12]. Tumor tissues were excised and fixed with 4% buffered formaldehyde. Fixed tissues were frozen in OCT compound, and sectioned at 5-μm thickness. After incubation with blocking buffer (5% bovine serum albumin, 0.01% triton-X100; 4°C, 60 min), slides were probed with anti-xCT or anti-γH2AX antibody (Santa Cruz Biotechnology) at 4°C overnight. The slides were then incubated with Alexa Fluor 488-conjugated anti-rabbit IgG secondary antibodies (1:2000, Thermo Fisher Scientific). After being washed twice with PBS, they were incubated with 300 nM 4′,6-diamidino-2-phenylindole for 20 min at room temperature, and mounted with ProLong Gold antifade reagent (Thermo Fisher Scientific). Fluorescent images were acquired using an BZ-X700 microscope.

### Experimental radiotherapy

When the tumor size was 100–200 mm^3^, SAS dissolved in PBS was intraperitoneally injected into tumor-bearing mice at a dose of 250 mg/kg/day for 3 sequential days. At 24 h after the third SAS administration, anesthetized tumor-bearing mice were locally X-irradiated at a dose of 4 Gy.

### Statistical analysis

All values are expressed as means ± standard deviations (SD) of three to five independent experiments. Differences between groups were evaluated by Student’s t-tests (two-sided) or Tukey-Kramer tests, and considered to be significant at p<0.05.

## Results

### SAS decreases cellular GSH and increases H_2_O_2_ cytotoxicity in B16F10 cells

**Fig 1:**
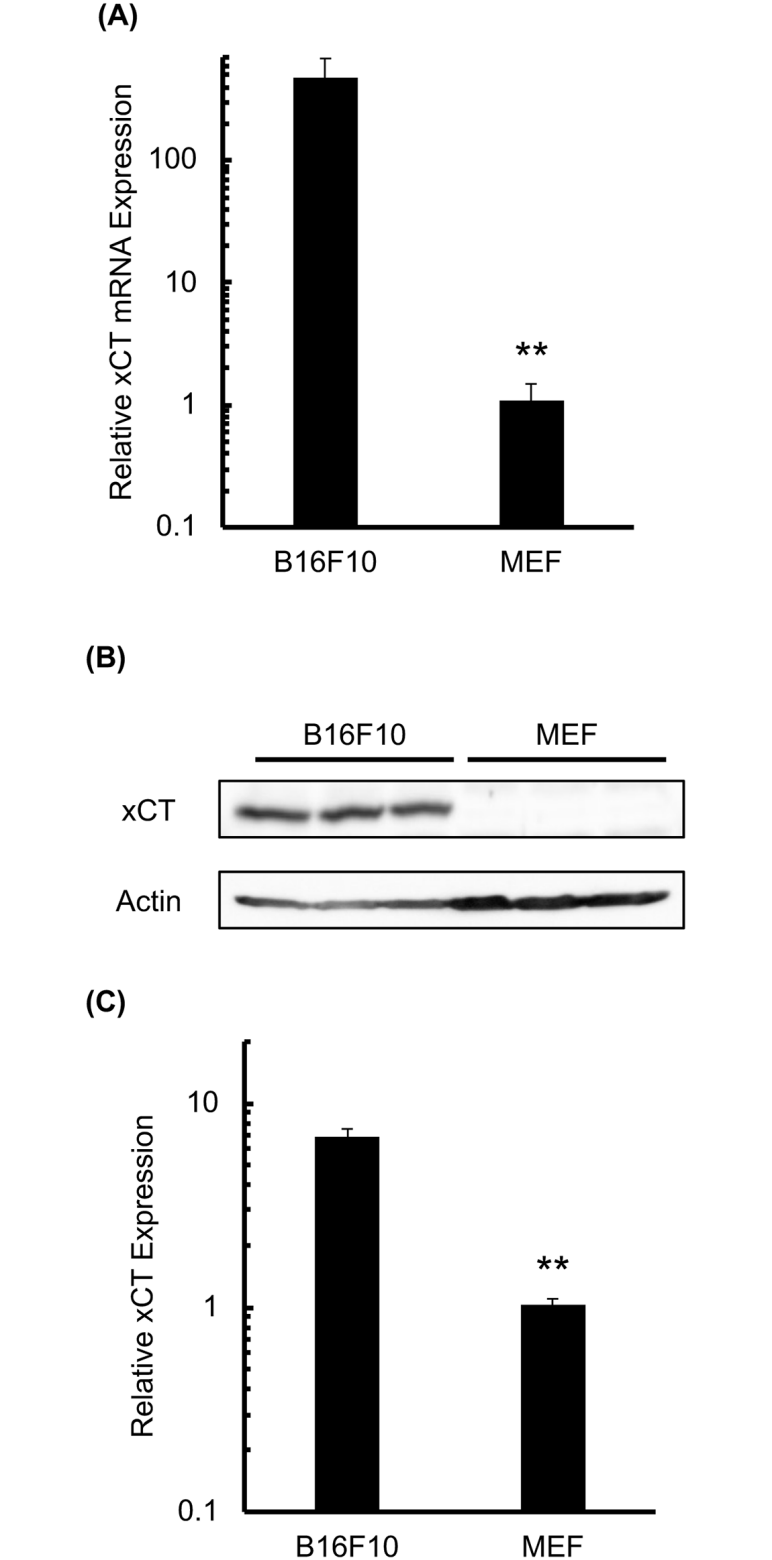
Murine B16F10 melanoma cells highly express xCT. B16F10 cells and MEF were subjected to RT-qPCR and western blot analyses of xCT expression. (A) RT-qPCR analysis for *xCT* mRNA expression in both cell types. (B) Image of a representative western blot for xCT in both cell types. (C) Western blot images were analyzed and the relative xCT expression was calculated for both cell types. Error bars = SD, *p<0.05.

*SLC7A11* gene codes for a sodium-independent cystine-glutamate antiporter that is chloride dependent, known as xCT. Firstly, *SLC7A11* gene expression was evaluated in B16F10 cells and MEF. RT-qPCR analysis revealed that *xCT* expression was 1,000-fold higher in B16F10 cells compared with MEF (Fig 1A). Furthermore, xCT protein was increased in B16F10 compared with MEF (Fig 1B and 1C).

**Fig 2:**
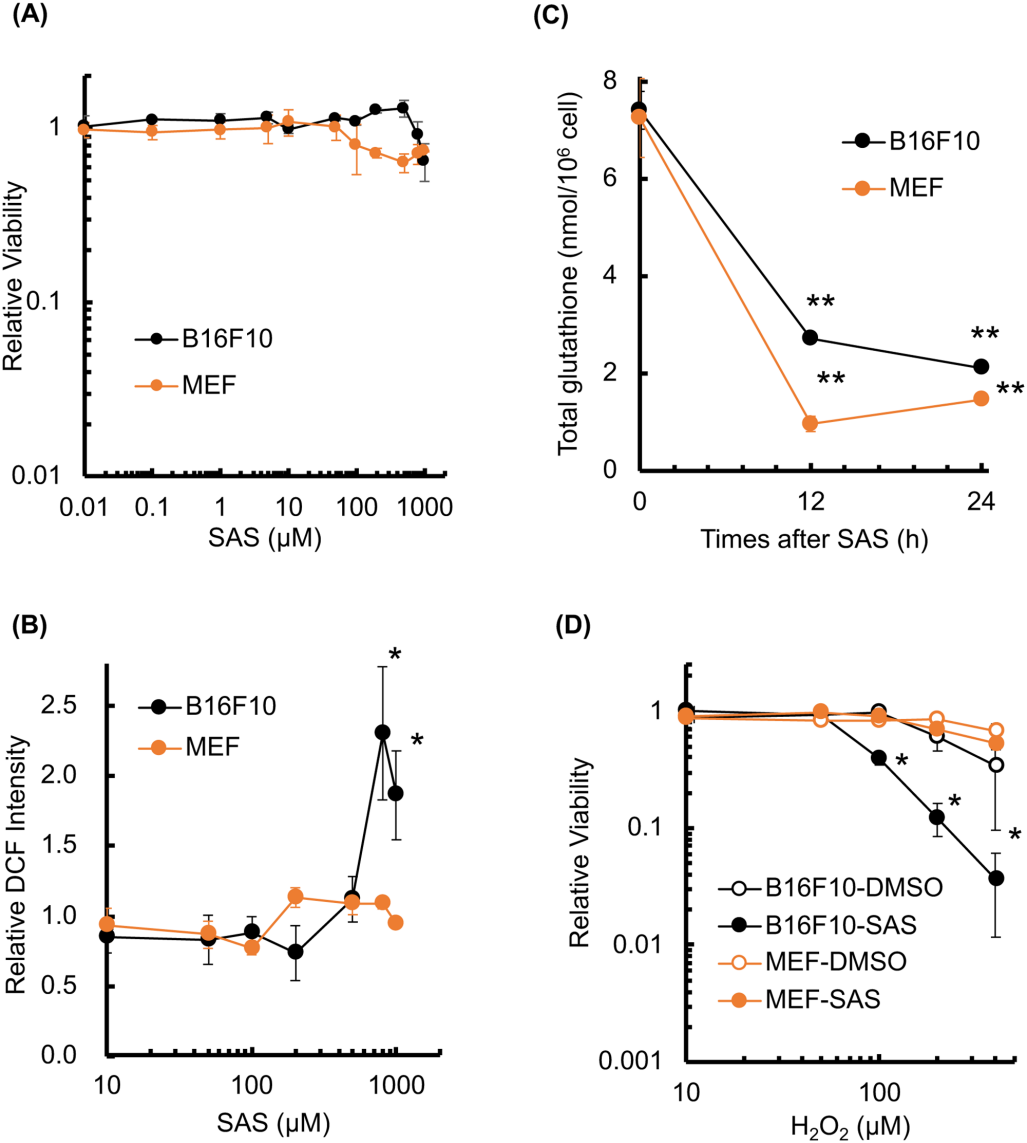
SAS decreases cellular resistance to H_2_O_2_. The cellular viability, GSH levels, ROS levels, and sensitivity to H_2_O_2_ were evaluated in B16F10 cells and MEF after SAS treatment. (A) Cellular viability after treatment with SAS (24 h). (B) Cellular ROS measurement after SAS treatment (24 h). ROS levels were analyzed by DCFDA staining. (C) Quantitative analysis of intracellular GSH concentration after SAS treatment (200 μM). (D) The effect of SAS on H_2_O_2_ cytotoxicity. Cells were treated with SAS (200 μM, 24 h) followed by treatment with H_2_O_2_ (24 h). Error bars = SD, *p<0.05.

Next, we assessed the cytotoxicity of SAS in both cell types. As shown Fig 2A, 0.01–100 μM SAS had little effect on cellular viability at 24 h after treatment, whereas 200–1,000 μM SAS slightly decreased the viability of both cells (Fig 2A). Cellular ROS and GSH levels were measured after SAS treatment. At lower SAS concentrations (10–100 μM), we did not observe any increase in intracellular ROS. At higher concentrations of SAS (800–1,000 μM), intracellular ROS increased approximately 2.3-fold in B16F10 cells (Fig 2B), whereas ROS did not increase in MEF cells (1.13±0.07-fold at 200 μM SAS). SAS significantly decreased intracellular GSH in both cells after 12 to 24 h (Fig 2C). In this condition, GSH/(GSH+GSSG) did not decrease after SAS treatment (S2 Fig).

Depletion of GSH decreases cellular sensitivity to exogenous oxidative stress. The effect of SAS on H_2_O_2_ cytotoxicity was analyzed in B16F10 cells and MEF. SAS enhanced H_2_O_2_ cytotoxicity In B16F10 cells (Fig 2D; DMSO half maximal inhibitory concentration, IC_50_ = 300 μM, SAS IC_50_ = 90 μM) but not in MEF (Fig 2D; DMSO IC_50_ = 800 μM, SAS IC_50_ = 800 μM). These results suggest that B16F10 cells are more sensitive to GSH depletion compared with MEF.

**Fig 3:**
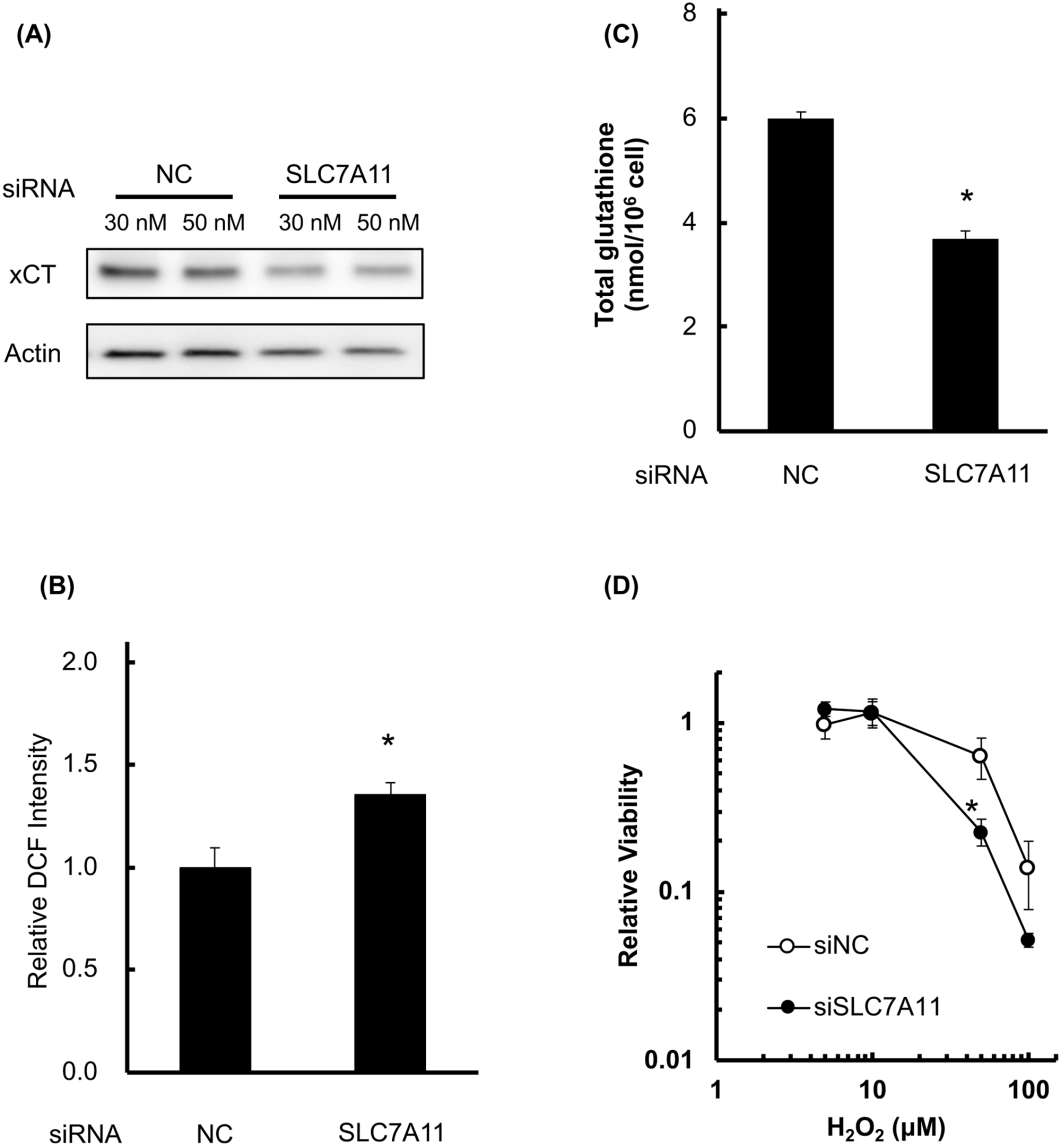
Knockdown of xCT decreases cellular resistance to H_2_O_2_. The cellular viability, GSH levels, ROS levels, and sensitivity to H_2_O_2_ were evaluated in B16F10 cells after siRNA treatment. (A) xCT expression after siSLC7A11 treatment. (B) Cellular ROS measurement after siRNA (72 h). ROS levels were analyzed by DCFDA staining. (C) Quantitative analysis of intracellular GSH concentration after siRNA. (D) The effect of SAS on H_2_O_2_ cytotoxicity. Cells were treated with siRNA (72 h) followed by treatment with H_2_O_2_ (24 h). Error bars = SD, *p<0.05.

Then, we analyzed the effect of RNA interference toward xCT on cellular GSH level, ROS, and H_2_O_2_ sensitivity (Fig 3). As shown in Fig 3A, RNA interference decreased xCT expression of B16F10 cells (30 nM; 51.8%, 50 nM; 69.4% compared to control siRNA condition), and we used 30 nM of siRNA in following examination. In this condition, GSH/GSSG ratio and total content of glutathione were decreased compared to scramble siRNA (Fig 3B). In this condition, GSH/(GSH+GSSG) did not decrease after SAS treatment (S2 Fig). With decrease of glutathione, cellular ROS level (Fig 3C) were increased and cellular sensitivity of H_2_O_2_ were increased in siRNA condition (Fig 3D).

### Radio-sensitizing effect of SAS on B16F10 cells

**Fig 4:**
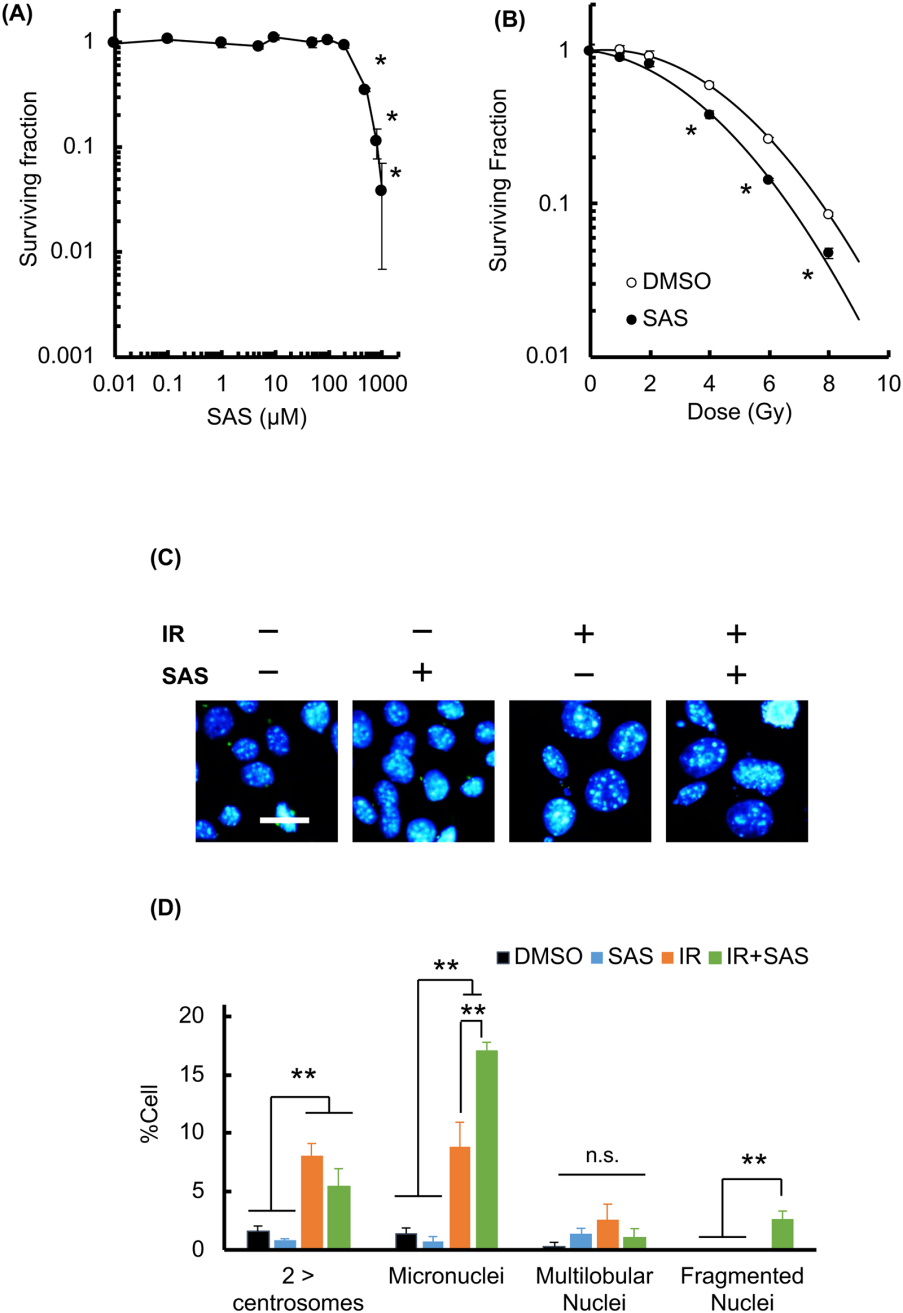
SAS sensitizes B16F10 cells to X-irradiation. A colony formation assay was used to determine the cytotoxicity and the radio-sensitizing effect of SAS treatment (24 h). (A) Colony formation assay for treatment with SAS alone. B16F10 cells were seeded and treated with SAS at the concentrations shown. (B) Colony formation assay for treatment with SAS plus X-irradiation. After pretreatment with SAS (200 μM, 24 h), B16F10 cells were subjected to X-irradiation at the doses shown. (C) Evaluation of MC fraction after X-irradiation. B16F10 cells were stained for γ-tubulin after 8 Gy of irradiation with or without SAS treatment. IR, X-irradiation. (D) Quantification of MC fractions after manual counting. IR, irradiation, error bars = SD, *p<0.05.

To examine whether radiation-induced cell death was enhanced by SAS treatment, a clonogenic survival assay was employed. B16F10 cells were pretreated with SAS for 24 h and exposed to X-rays in the presence of SAS. Treatment with SAS gradually suppressed cellular survival (Fig 4A). Survival curves are shown in Fig 4B. The 10% lethal doses were 7.8 Gy for controls and 6.75 Gy with SAS treatment (sensitizer enhancement ratio = 1.55). To clarify the effect of SAS on radiation-induced cell death, the fraction of cells undergoing MC, a marker of reproductive death, was evaluated. The nuclei and centrosomes of irradiated cells were stained, and the numbers of cells showing markers of MC, including aberrant mitotic nuclei (micronuclei, multi-lobular nuclei, and fragmented nuclei) and more than two centrosomes (Fig 4C) were scored. As shown in Fig 4D, MC was significantly increased at 24 h after 8 Gy of irradiation, and SAS synergistically increased MC in irradiated cells, especially formation of micronuclei and fragmented nuclei. Under these conditions, we did not observe that SAS increased radiation-induced apoptosis (data not shown). Therefore, SAS enhanced MC after X-irradiation, leading to radio-sensitization of B16F10 cells.

### SAS treatment decreases DNA double strand break repair capacity after X-irradiation

**Fig 5:**
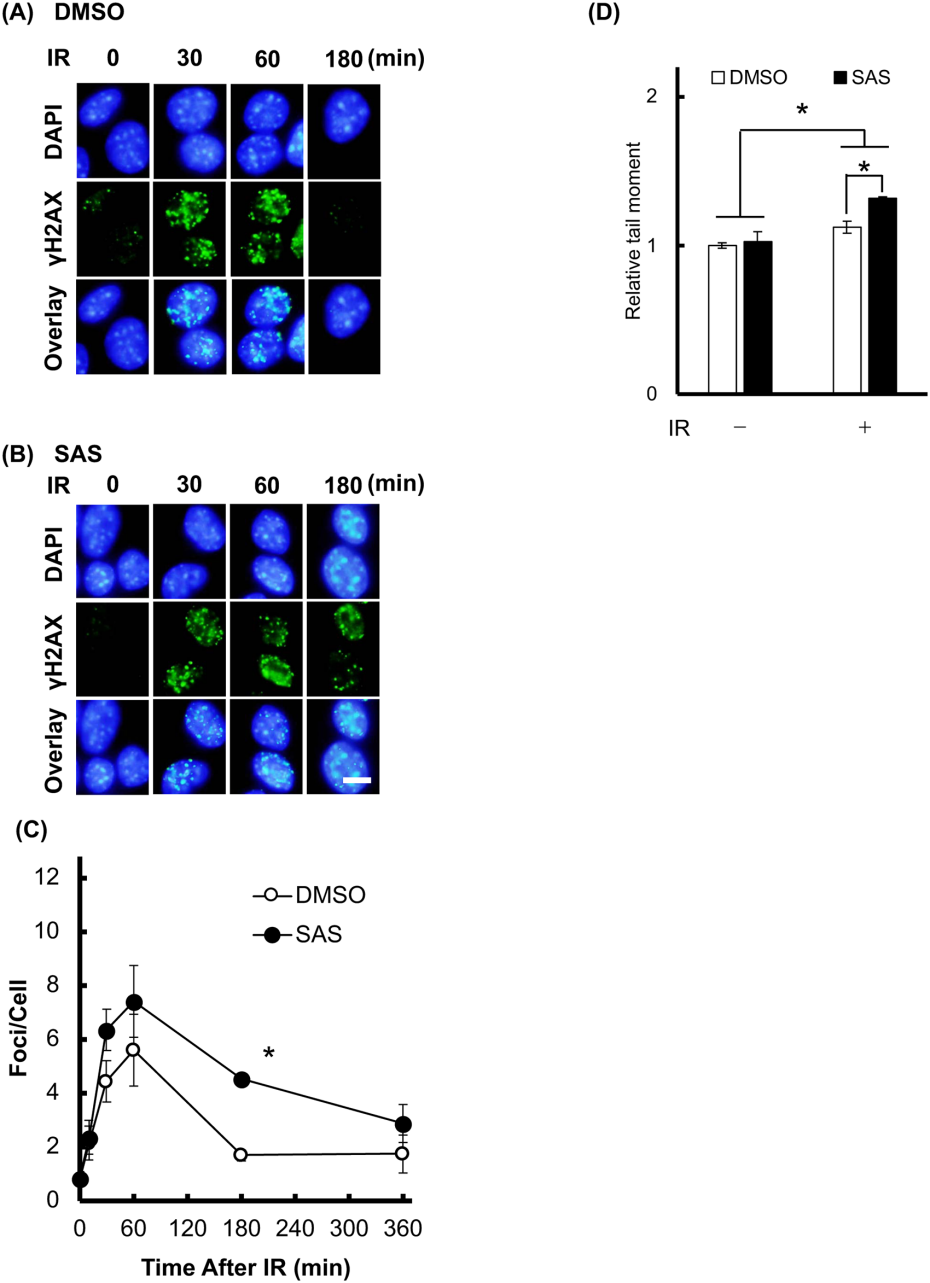
SAS inhibits cellular DNA damage repair. The effect of SAS on DNA damage repair capacity was evaluated in B16F10 cells after X-irradiation at a dose of 1 Gy (IR). At the time points shown in figure, cells were immunostained for the DSB marker, γH2AX. Representative images of γH2AX foci after irradiation are shown, following pretreatment with (A) DMSO or (B) SAS. Scale bar = 10 μm. (C) Quantification of the numbers of γH2AX foci. (D) Relative tail moment was analyzed by comet assay at 60 min after 1 Gy of X-irradiation. Error bars = SD, *p<0.05.

It has been demonstrated that MC in mammalian cells results from the failure of mitosis involving incomplete repair of DNA damage [20]. To explore the involvement of cellular DNA damage repair in SAS-induced radio-sensitization, we analyzed double strand break (DSB; γH2AX staining) formation in irradiated cells [21]. When the cells were irradiated at 1 Gy without SAS, the number of DSB peaked at 5.6±1.3 foci per cell at 60 min after X-irradiation, and then decreased at later time points (Fig 5A–5C) until most DSB had been repaired at 180 min (1.69±0.2 foci per cell). However, pretreatment with SAS significantly inhibited the decrease in the number of foci at 180 min (4.5±0.7). At 60 min after irradiation, SAS have tendency to increase the number of γH2AX foci. In addition, knockdown of xCT by siRNA transfection, and knockdown of xCT increased γH2AX after X-irradiation (S3 Fig). To clarify the effect of SAS on primary cytotoxicity toward X-irradiation, we employed alkaline comet assay to analyze formation of strand breaks after X-irradiation. As shown Fig 5D, SAS treatment increased the comet tail moment.

**Fig 6:**
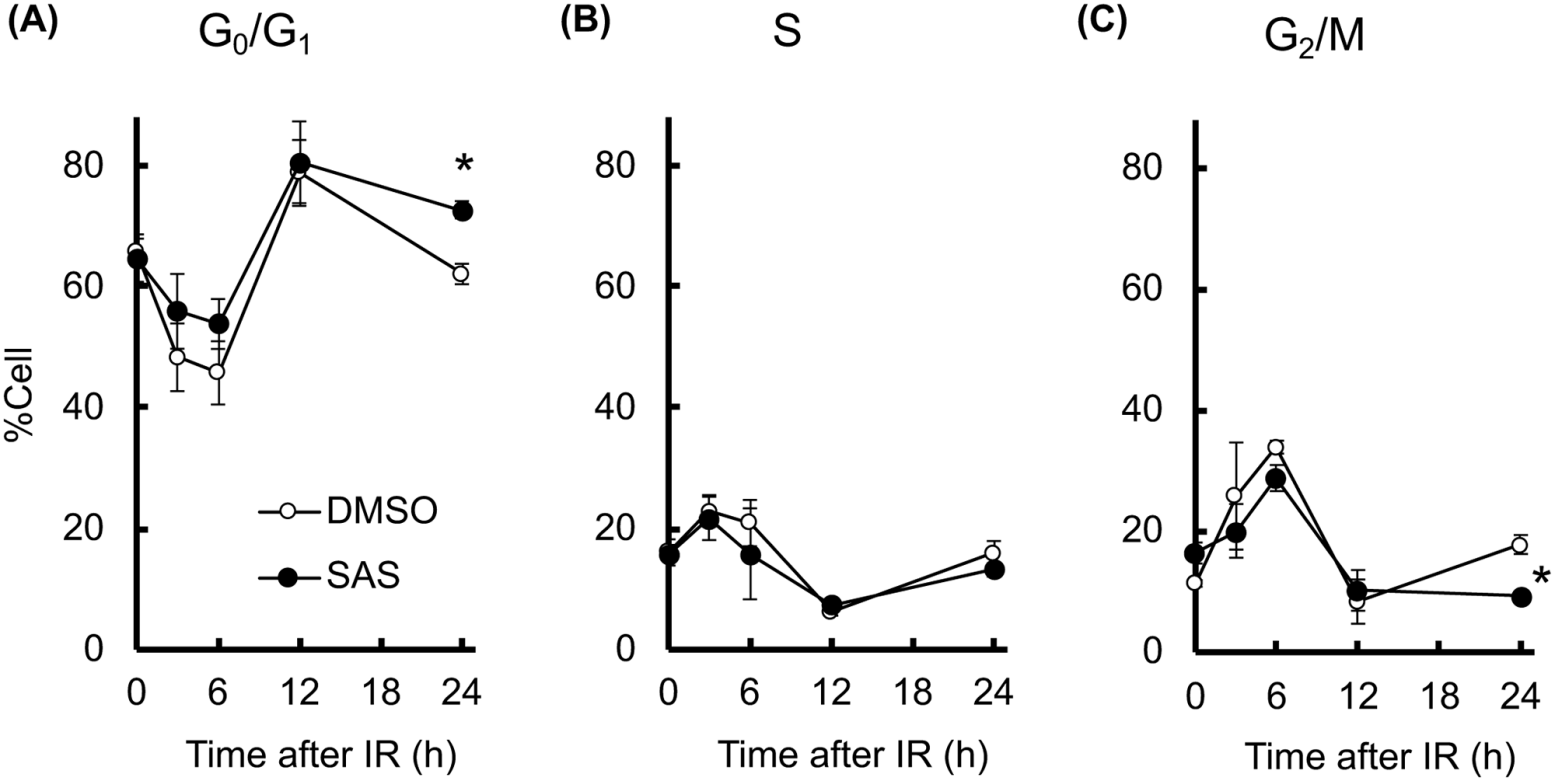
SAS prolongs cell cycle arrest. The effect of SAS on cell cycle perturbation after X-irradiation (IR) was determined. Populations of (A) G_0_/G_1_-phase, (B) S-phase, and (C) G2/M-phase were analyzed by propidium iodide staining and flow cytometry. Error bars = SD, *p<0.05.

Attenuation of DNA damage repair may influence the cell cycle check point and induce prolonged cell cycle arrest [22]. We therefore investigated the effect of SAS on cell cycle arrest after X-irradiation (Fig 6). The population of G_0_/G_1_-phase cells significantly increased at 12 h after X-irradiation. Control G_0_/G_1_-phase cells then decreased to baseline levels at 24 h, whereas SAS pretreatment induced prolonged G_0_/G_1_ arrest (Fig 6A). Cell numbers in S-phase (Fig 6B) and G_2_/M-phase (Fig 6C) were also perturbed by X-irradiation, with control cells returning to baseline levels after 24 h, while SAS pretreatment decreased G_2_/M-phase cells at 24 h (Fig 6C). Supporting this result, knockdown of xCT induces these perturbations of cell cycle after X-irradiation (S4 Fig). Therefore, depletion of GSH by SAS attenuated cellular DSB repair capacity and enhanced G_0_/G_1_ arrest after X-irradiation.

### Radio-enhancement effect of SAS on B16F10 allograft tumors

**Fig 7:**
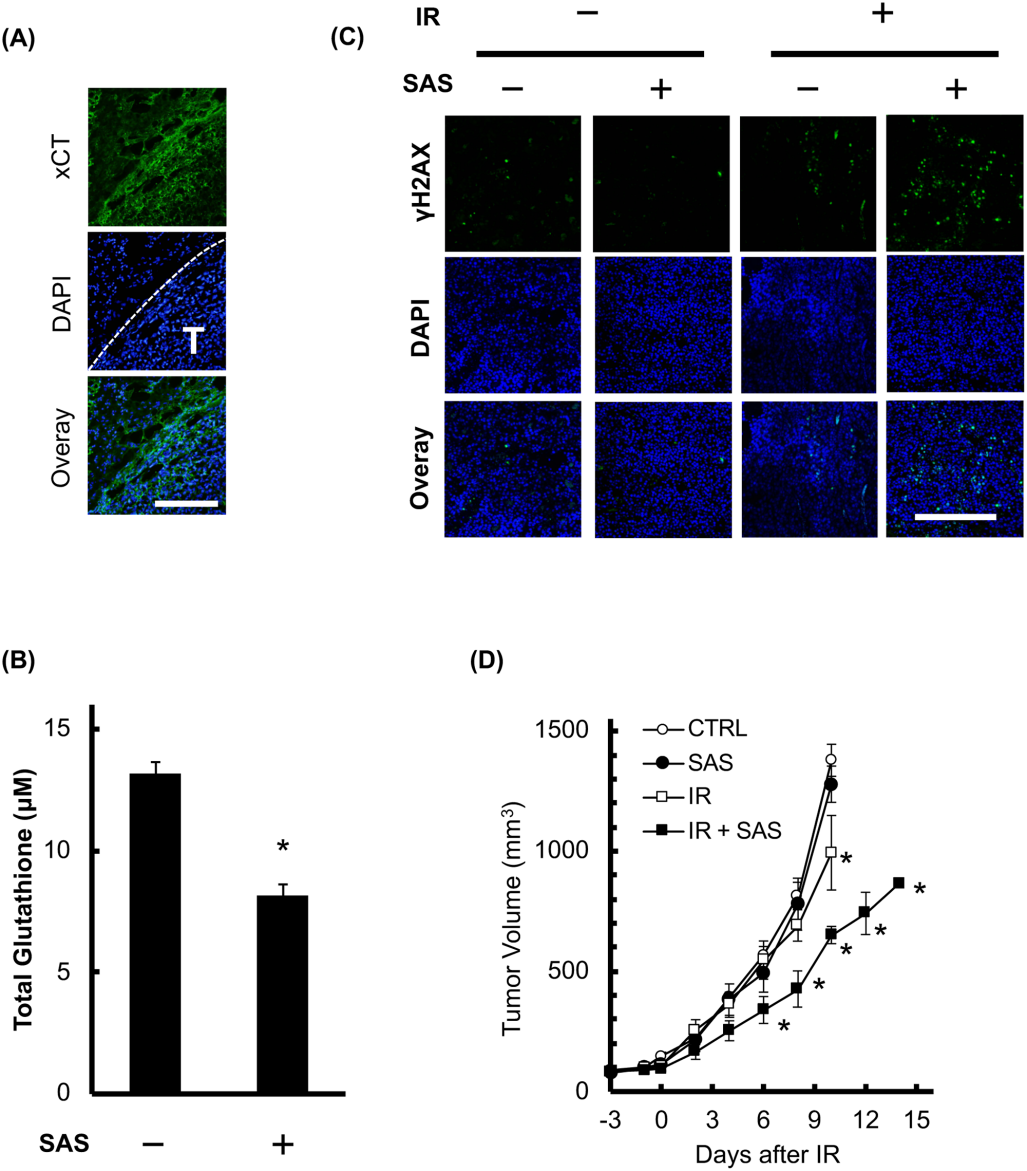
SAS sensitizes transplanted B16F10 tumors to irradiation. The radio-sensitizing effect of SAS was evaluated on tumors formed by B16F10 cells inoculated into the hind legs of C57BL/6N mice. (A) Images of xCT immunostaining in a representative tumor. The transplanted B16F10 tumor was excised and immunostained for xCT. White T, tumor region; scale bar = 200 μm. (B) Intratumoral GSH concentration with and without SAS treatment. After 12 days, transplanted B16F10 tumors were treated with SAS (24 h, 250 mg/kg intraperitoneally). Error bars = SD, *p<0.05. (C) Representative images of γH2AX staining at 3 h after 1 Gy X-irradiation with or without pretreatment with SAS. IR, irradiation, scale bar = 200 μm. (D) Experimental radiotherapy to validate the effect of SAS. B16F10 tumor-bearing mice were treated with SAS for 3 days at 24 h intervals, and then X-irradiated at a dose of 4 Gy (n = 4). Tumor volumes were measured as described in the Materials and Methods section. IR, irradiation, error bars = SD, *p<0.05.

Since SAS decreased intracellular GSH and enhanced radiosensitivity *in vitro*, we evaluated the effect of SAS on transplanted B16F10 melanomas. Immunohistochemistry of xCT showed enhanced expression in B16F10 tumors and low expression in surrounding skin (Fig 7A and S5 Fig). SAS treatment significantly decreased the intratumoral GSH concentration at 24 h after administration (Fig 7B). We also evaluated the number of γH2AX-positive nuclei after X-irradiation by immunohistochemistry. X-irradiation at 1 Gy slightly increased the number of γH2AX-positive cells, and pretreatment with SAS drastically increased them (Fig 7C). Next, we investigated the effect of SAS treatment on experimental radiotherapy-induced tumor growth delay. As shown in Fig 7D, the tumor volume without treatment increased more than 9.4-fold over 10 days (to 1377.8±163.3 mm^3^), and SAS alone induced no significant inhibition of tumor growth (1278.4±219.1 mm^3^ at 10 days). X-irradiation partially decreased tumor growth (993.6±168.2 mm^3^ at 10 days). Combination therapy of SAS with X-irradiation synergistically decreased tumor growth (651.1±71.9 mm^3^ at 10 days). These results suggest that SAS treatment sensitized tumors to radiotherapy in the B16F10 cell model.

## Discussion

Melanoma is considered to be a radio-resistant cancer, and the multiple mechanisms underlying radio-resistance are incompletely characterized [23]. In some tumor cell lines, radio-resistance is mediated by increased synthesis of GSH leading to the scavenging of ROS produced by irradiation [23, 24]. Intracellular GSH is the major component of non-protein thiol and acts to resist oxidative stress in normal tissue. However, in cancer tissue, various reports have suggested that GSH is involved in chemo-/radio-therapy resistance, and have demonstrated that GSH depletion increases the efficacy of chemo- and radio-therapy [9, 25, 26]. Since xCT plays a pivotal role in the cellular uptake of cystine [3], inhibition of xCT may contribute to optimizing cancer therapy.

The xCT inhibitor, SAS was first synthesized by combining sulfapyridine, an antibiotic, with 5-aminosalicylic acid, an anti-inflammatory agent, linked by an azo bridge. It is commonly used to treat chronic inflammatory diseases and rheumatoid arthritis [27, 28]. SAS is usually administered orally and approximately 70% of the drug is degraded by colonic bacteria via azo-cleavage to sulfapyridine and 5-aminosalicylic acid [29]. These studies suggested the possibility of SAS acting as an anticancer agent based on its inhibition of xCT. However, its molecular mechanism and tumor selectivity have not been elucidated.

In this study, xCT expression was significantly higher in B16F10 cells compared with normal cells (Figs 1B, 7A, and S5 Fig), and SAS treatment significantly decreased the intracellular GSH concentration in both B16F10 and normal cells. However, SAS treatment and xCT knockdown increased intracellular ROS levels and H_2_O_2_ toxicity in B16F10 but not in normal cells (Figs 2 and 3). These results explain the dependency of melanoma cells on the reductase activity of GSH and the specific radio-sensitization effect of SAS on B16F10 melanoma cells compared to MEF. As shown in Figs 4B and 7C, pretreatment with SAS increased the sensitivity of B16F10 tumors to X-irradiation. To clarify the mechanism of the radio-sensitizing effect of SAS, we evaluated its effects on DNA damage repair and cell cycle perturbation after X-irradiation. Pretreatment with SAS diminished DSB repair and prolonged G1 arrest (Figs 5, 6 and 7C). Depletion of GSH by SAS may decrease cellular DNA damage repair leading to MC after X-irradiation (Fig 4C and 4D). RNA interference toward xCT decreased DSB repair and prolonged G1 arrest after X-irradiation (S2 and S3 Figs). Therefore, GSH depletion by SAS decreases DSB repair capacity and sensitizes B16F10 melanomas to X-irradiation. We did not employ MEF cells as low xCT expression cell along with siRNA condition because of radiation-induced senescence of primary cells [30, 31].

In addition to attenuation of DSB repair, GSH depletion might enhance chorionic effect of X-irradiation. As shown Figs 5 and 6, G_1_ arrest occurs at 24 hours after X-irradiation despite DSBs are almost repaired in 6 h after irradiation. G_1_ cell cycle arrest can be induced by cellular senescence, ATP level, and genomic instability, and ionizing radiation can affect these cellular events [32, 33]. Thus, GSH depletion might enhances the chronic effect of X-irradiation as well as attenuation of DSB repair.

Antioxidant systems allow cancer stem cells to avoid the adverse consequences of oxidative stress. Therefore, inhibition of GSH synthesis by SAS attenuates the radio-resistance of cancer stem cells [34]. CD44v is a major marker for cancer stem cells in many epithelial tumors and is implicated in tumor growth, invasion and metastasis [7]. Wada *et al*. have shown that the interaction of CD44v with xCT stabilizes the latter protein and thereby potentiates the ability of cancer cells to defend themselves against ROS [34]. Yoshikawa *et al*. have also demonstrated that SAS treatment attenuated CD44v-expressing cancer stem cells [35]. In addition, Rodman *et al*. showed an enhanced response of breast cancer stem cells to SAS combined with chemo/radiotherapy [9]. Because of the radio-resistant phenotype of cancer stem cells, these studies suggest the clinical possibility of using SAS as a radio-sensitizer to target cancer stem cells. In addition to our proposing mechanism, other mechanism may involve in radio-sensitizing effect. Recent study has shown the role of xCT on tumor hypoxia [36]. Furthermore, cystin and cysteine are not only substrate for glutathione but also reduction protein synthesis such as thioredoxin.

Our present data provide evidence of the radio-sensitizing effect of SAS in a murine melanoma not in normal cell, and furthers understanding of the underlying molecular mechanisms. Since further studies are needed to validate the effect of SAS as a radio-sensitizer in spontaneous tumors, we are conducting a veterinary clinical trial on spontaneous canine melanoma.

## Supporting information

S1 Text Supplementary materials and methods. (DOCX)

S1 Table NC3Rs ARRIVE guidelines checklist. (PDF)

S1 Fig Body weight of tumor bearing mice. Body weight were measured after tumor transplantation.
(TIF)

S2 Fig Inhibition of xCT did not change the GSH/(GSH+GSSG) ratio. GSH/(GSH+GSSG) were analyzed after xCT inhibition. (A) Cells were treated by SAS (200 μM, 24 h) and analyzed GSH+GSSG concentration and GSSG concentration. (B) Cells were treated by siSLC7A11 (30 nM, 72 h) and analyzed GSH and GSSG ratio.
(TIF)

S3 Fig Knock down of xCT decrease DSB repair capacity. Cells were treated by siSLC7A11 (30 nM, 72 h) and irradiated at dose of 1 Gy. Then, cells were stained by phospho-H_2_AX. (A) representative image of phospho-H_2_AX after X-irradiation. White bar = 10 μM. (B) quantitative results of H_2_AX foci formation. Error Bar = S.D., p* < 0.05.
(TIF)

S4 Fig Knock down of xCT enhances radiation induced cell cycle arrest. Cells were treated by siSLC7A11 (30 nM, 72 h) and irradiated at dose of 1 Gy. Then, cells were fixed at indicated time. Populations of (A) G_0_/G_1_-phase, (B) S-phase, and (C) G2/M-phase were analyzed by propidium iodide staining and flow cytometry. Error bars = SD, *p<0.05.
(TIF)

S5 Fig Advanced expression of xCT on B16F10 tumor. B16F10 tumors were excised and lysed in RIPA buffer. Expression of xCT were measured by western blot. (A) image of western blot for 6 tumors and 6 skin tissues. (B) Quantitative analysis of band intensity of xCT. Bar = S.D., p* < 0.05.
(TIF)

S6 Fig Western blot image. (TIF)

## References

[1] Chabner B, LL D. Cancer chemotherapy and biotherapy: principles and practice. 5th ed: Lippincott Williams & Wilkins; 2011.

[2] Lee KS, Kim HK, Moon HS, Hong YS, Kang JH, Kim DJ, et al Effects of buthionine sulfoximine treatment on cellular glutathione levels and cytotoxicities of cisplatin, carboplatin and radiation in human stomach and ovarian cancer cell lines. Korean J Intern Med. 1992;7(2):111–7. doi: 10.3904/kjim.1992.7.2.111 1306072 10.3904/kjim.1992.7.2.111 PMC4532113

[3] Bridges CC, Kekuda R, Wang H, Prasad PD, Mehta P, Huang W, et al Structure, function, and regulation of human cystine/glutamate transporter in retinal pigment epithelial cells. Invest Ophthalmol Vis Sci. 2001;42(1):47–54. . 11133847

[4] Xie L, Song X, Yu J, Guo W, Wei L, Liu Y, et al Solute carrier protein family may involve in radiation-induced radioresistance of non-small cell lung cancer. J Cancer Res Clin Oncol. 2011;137(12):1739–47. doi: 10.1007/s00432-011-1050-9 . 21909646 10.1007/s00432-011-1050-9 PMC11828279

[5] Toyoda M, Kaira K, Ohshima Y, Ishioka NS, Shino M, Sakakura K, et al Prognostic significance of amino-acid transporter expression (LAT1, ASCT2, and xCT) in surgically resected tongue cancer. Br J Cancer. 2014;110(10):2506–13. doi: 10.1038/bjc.2014.178 24762957 10.1038/bjc.2014.178 PMC4021522

[6] Gout PW, Buckley AR, Simms CR, Bruchovsky N. Sulfasalazine, a potent suppressor of lymphoma growth by inhibition of the x(c)- cystine transporter: a new action for an old drug. Leukemia. 2001;15(10):1633–40. . 11587223 10.1038/sj.leu.2402238

[7] Ishimoto T, Nagano O, Yae T, Tamada M, Motohara T, Oshima H, et al CD44 variant regulates redox status in cancer cells by stabilizing the xCT subunit of system xc(-) and thereby promotes tumor growth. Cancer Cell. 2011;19(3):387–400. doi: 10.1016/j.ccr.2011.01.038 . 21397861 10.1016/j.ccr.2011.01.038

[8] Sleire L, Skeie BS, Netland IA, Forde HE, Dodoo E, Selheim F, et al Drug repurposing: sulfasalazine sensitizes gliomas to gamma knife radiosurgery by blocking cystine uptake through system Xc-, leading to glutathione depletion. Oncogene. 2015;34(49):5951–9. doi: 10.1038/onc.2015.60 . 25798841 10.1038/onc.2015.60

[9] Rodman SN, Spence JM, Ronnfeldt TJ, Zhu Y, Solst SR, O’Neill RA, et al Enhancement of Radiation Response in Breast Cancer Stem Cells by Inhibition of Thioredoxin- and Glutathione-Dependent Metabolism. Radiat Res. 2016;186(4):385–95. doi: 10.1667/RR14463.1 27643875 10.1667/RR14463.1 PMC5077643

[10] Nishida N, Yasui H, Nagane M, Yamamori T, Inanami O. 3-Methyl pyruvate enhances radiosensitivity through increasing mitochondria-derived reactive oxygen species in tumor cell lines. J Radiat Res. 2014;55(3):455–63. doi: 10.1093/jrr/rrt142 . 24385472 10.1093/jrr/rrt142 PMC4014165

[11] Sakai Y, Yamamori T, Yasui H, Inanami O. Downregulation of the DNA repair enzyme apurinic/apyrimidinic endonuclease 1 stimulates transforming growth factor-beta1 production and promotes actin rearrangement. Biochem Biophys Res Commun. 2015;461(1):35–41. doi: 10.1016/j.bbrc.2015.03.163 . 25858321 10.1016/j.bbrc.2015.03.163

[12] Nagane M, Yasui H, Yamamori T, Zhao S, Kuge Y, Tamaki N, et al Radiation-induced nitric oxide mitigates tumor hypoxia and radioresistance in a murine SCCVII tumor model. Biochemical and Biophysical Research Communications. 2013;437(3):420–5. doi: 10.1016/j.bbrc.2013.06.093 23831468 10.1016/j.bbrc.2013.06.093

[13] Nagane M, Yasui H, Sakai Y, Yamamori T, Niwa K, Hattori Y, et al Activation of eNOS in endothelial cells exposed to ionizing radiation involves components of the DNA damage response pathway. Biochem Biophys Res Commun. 2015;456(1):541–6. doi: 10.1016/j.bbrc.2014.12.002 . 25498542 10.1016/j.bbrc.2014.12.002

[14] Yasui H, Yamamoto K, Suzuki M, Sakai Y, Bo T, Nagane M, et al Lipophilic triphenylphosphonium derivatives enhance radiation-induced cell killing via inhibition of mitochondrial energy metabolism in tumor cells. Cancer Lett. 2017;390:160–7. doi: 10.1016/j.canlet.2017.01.006 . 28093283 10.1016/j.canlet.2017.01.006

[15] Yamamori T, Ike S, Bo T, Sasagawa T, Sakai Y, Suzuki M, et al Inhibition of the mitochondrial fission protein dynamin-related protein 1 (Drp1) impairs mitochondrial fission and mitotic catastrophe after x-irradiation. Mol Biol Cell. 2015;26(25):4607–17. doi: 10.1091/mbc.E15-03-0181 26466676 10.1091/mbc.E15-03-0181 PMC4678018

[16] Yasui H, Takeuchi R, Nagane M, Meike S, Nakamura Y, Yamamori T, et al Radiosensitization of tumor cells through endoplasmic reticulum stress induced by PEGylated nanogel containing gold nanoparticles. Cancer Lett. 2014 doi: 10.1016/j.canlet.2014.02.005 . 24530512 10.1016/j.canlet.2014.02.005

[17] Olive PL, Banath JP. The comet assay: a method to measure DNA damage in individual cells. Nat Protoc. 2006;1(1):23–9. doi: 10.1038/nprot.2006.5 . 17406208 10.1038/nprot.2006.5

[18] Gyori BM, Venkatachalam G, Thiagarajan PS, Hsu D, Clement MV. OpenComet: an automated tool for comet assay image analysis. Redox Biol. 2014;2:457–65. doi: 10.1016/j.redox.2013.12.020 24624335 10.1016/j.redox.2013.12.020 PMC3949099

[19] Yasui H, Ogura A, Asanuma T, Matsuda A, Kashiwakura I, Kuwabara M, et al Inhibition of HIF-1alpha by the anticancer drug TAS106 enhances X-ray-induced apoptosis in vitro and in vivo. Br J Cancer. 2008;99(9):1442–52. doi: 10.1038/sj.bjc.6604720 18854835 10.1038/sj.bjc.6604720 PMC2579694

[20] Castedo M, Perfettini JL, Roumier T, Andreau K, Medema R, Kroemer G. Cell death by mitotic catastrophe: a molecular definition. Oncogene. 2004;23(16):2825–37. doi: 10.1038/sj.onc.1207528 . 15077146 10.1038/sj.onc.1207528

[21] Kurashige T, Shimamura M, Nagayama Y. Differences in quantification of DNA double-strand breaks assessed by 53BP1/gammaH2AX focus formation assays and the comet assay in mammalian cells treated with irradiation and N-acetyl-L-cysteine. J Radiat Res. 2016;57(3):312–7. doi: 10.1093/jrr/rrw001 26951077 10.1093/jrr/rrw001 PMC4915540

[22] Ciccia A, Elledge SJ. The DNA damage response: making it safe to play with knives. Mol Cell. 2010;40(2):179–204. doi: 10.1016/j.molcel.2010.09.019 20965415 10.1016/j.molcel.2010.09.019 PMC2988877

[23] Pak BJ, Lee J, Thai BL, Fuchs SY, Shaked Y, Ronai Z, et al Radiation resistance of human melanoma analysed by retroviral insertional mutagenesis reveals a possible role for dopachrome tautomerase. Oncogene. 2004;23(1):30–8. doi: 10.1038/sj.onc.1207007 . 14712208 10.1038/sj.onc.1207007

[24] Hall EJ, Giaccia AJ. Radiobiology for the Radiologist. 7th ed Philadelphia (PA): Lippincott Williams & Wilkins; 2011.

[25] Clark EP, Epp ER, Biaglow JE, Morse-Gaudio M, Zachgo E. Glutathione depletion, radiosensitization, and misonidazole potentiation in hypoxic Chinese hamster ovary cells by buthionine sulfoximine. Radiat Res. 1984;98(2):370–80. . 6539482

[26] Tagde A, Singh H, Kang MH, Reynolds CP. The glutathione synthesis inhibitor buthionine sulfoximine synergistically enhanced melphalan activity against preclinical models of multiple myeloma. Blood Cancer J. 2014;4:e229 doi: 10.1038/bcj.2014.45 25036800 10.1038/bcj.2014.45 PMC4219442

[27] Klotz U, Maier K, Fischer C, Heinkel K. Therapeutic efficacy of sulfasalazine and its metabolites in patients with ulcerative colitis and Crohn’s disease. The New England journal of medicine. 1980;303(26):1499–502. doi: 10.1056/NEJM198012253032602 . 6107853 10.1056/NEJM198012253032602

[28] O’Dell JR, Leff R, Paulsen G, Haire C, Mallek J, Eckhoff PJ, et al Treatment of rheumatoid arthritis with methotrexate and hydroxychloroquine, methotrexate and sulfasalazine, or a combination of the three medications: results of a two-year, randomized, double-blind, placebo-controlled trial. Arthritis Rheum. 2002;46(5):1164–70. doi: 10.1002/art.10228 . 12115219 10.1002/art.10228

[29] Guastavino E, Litwin NH, Heffes Nahmod L, Licastro R. Ulcerative colitis in children. Levels of salicylazosulfapyridine and sulfapyridine during treatment. Acta Gastroenterol Latinoam. 1988;18(2):107–13. . 2908013

[30] Day RM, Snow AL, Panganiban RA. Radiation-induced accelerated senescence: a fate worse than death? Cell cycle. 2014;13(13):2011–2. doi: 10.4161/cc.29457 24922064 10.4161/cc.29457 PMC4111686

[31] Marthandan S, Menzel U, Priebe S, Groth M, Guthke R, Platzer M, et al Conserved genes and pathways in primary human fibroblast strains undergoing replicative and radiation induced senescence. Biol Res. 2016;49(1):34 doi: 10.1186/s40659-016-0095-2 27464526 10.1186/s40659-016-0095-2 PMC4963952

[32] Di Leonardo A, Linke SP, Clarkin K, Wahl GM. DNA damage triggers a prolonged p53-dependent G1 arrest and long-term induction of Cip1 in normal human fibroblasts. Genes Dev. 1994;8(21):2540–51. . 7958916 10.1101/gad.8.21.2540

[33] Moiseeva O, Mallette FA, Mukhopadhyay UK, Moores A, Ferbeyre G. DNA damage signaling and p53-dependent senescence after prolonged beta-interferon stimulation. Mol Biol Cell. 2006;17(4):1583–92. doi: 10.1091/mbc.E05-09-0858 16436515 10.1091/mbc.E05-09-0858 PMC1415317

[34] Wada T, Ishimoto T, Seishima R, Tsuchihashi K, Yoshikawa M, Oshima H, et al Functional role of CD44v-xCT system in the development of spasmolytic polypeptide-expressing metaplasia. Cancer Sci. 2013;104(10):1323–9. doi: 10.1111/cas.12236 . 23848514 10.1111/cas.12236 PMC7656553

[35] Yoshikawa M, Tsuchihashi K, Ishimoto T, Yae T, Motohara T, Sugihara E, et al xCT inhibition depletes CD44v-expressing tumor cells that are resistant to EGFR-targeted therapy in head and neck squamous cell carcinoma. Cancer Res. 2013;73(6):1855–66. doi: 10.1158/0008-5472.CAN-12-3609-T . 23319806 10.1158/0008-5472.CAN-12-3609-T

[36] Lu H, Samanta D, Xiang L, Zhang H, Hu H, Chen I, et al Chemotherapy triggers HIF-1-dependent glutathione synthesis and copper chelation that induces the breast cancer stem cell phenotype. Proc Natl Acad Sci U S A. 2015;112(33):E4600–9. doi: 10.1073/pnas.1513433112 26229077 10.1073/pnas.1513433112 PMC4547233

