# Supplementary material for: Sulfasalazine, an inhibitor of the cystine-glutamate antiporter, reduces DNA damage repair and enhances radiosensitivity in murine B16F10 melanoma
Source: PLoS One. 2018 Apr 12;13(4):e0195151. doi: 10.1371/journal.pone.0195151 (PMC5896924; doi:10.1371/journal.pone.0195151)
Supplement: S1 Text — (DOCX) [file pone.0195151.s001.docx]

**SUPPLEMENTARY MATERIALS AND METHODS**

**GSH/(GSH+GSSG) measurement**

GSH/(GSH+GSSG) ratio was measured using a GSSG/GSH Quantification Kit (Cat No. G257; Dojindo) according to manufacturing instraction. Glutahione concentrations were calculated from absorbance at 405 nm and a GSH standard curve, and GSSG/GSH were calculated from concentrations of total glutathione (GSH+GSSG) and GSH.

**Western Blotting for tissue**

Tumor and skin tissues were lysed in modified RIPA buffer using an bead beater-type homogenizer (µT-12, Taitec corporation, Saitama, Japan). Lysed cell and tissue were centrifuged at 15,000×g for 15 min at 4°C, and the supernatants were collected as protein samples. SDS-PAGE and western blotting were performed as described in materials and methods section.
