# Supplementary figures and images for: Sulfasalazine, an inhibitor of the cystine-glutamate antiporter, reduces DNA damage repair and enhances radiosensitivity in murine B16F10 melanoma
Source: PLoS One. 2018 Apr 12;13(4):e0195151. doi: 10.1371/journal.pone.0195151 (PMC5896924; doi:10.1371/journal.pone.0195151)

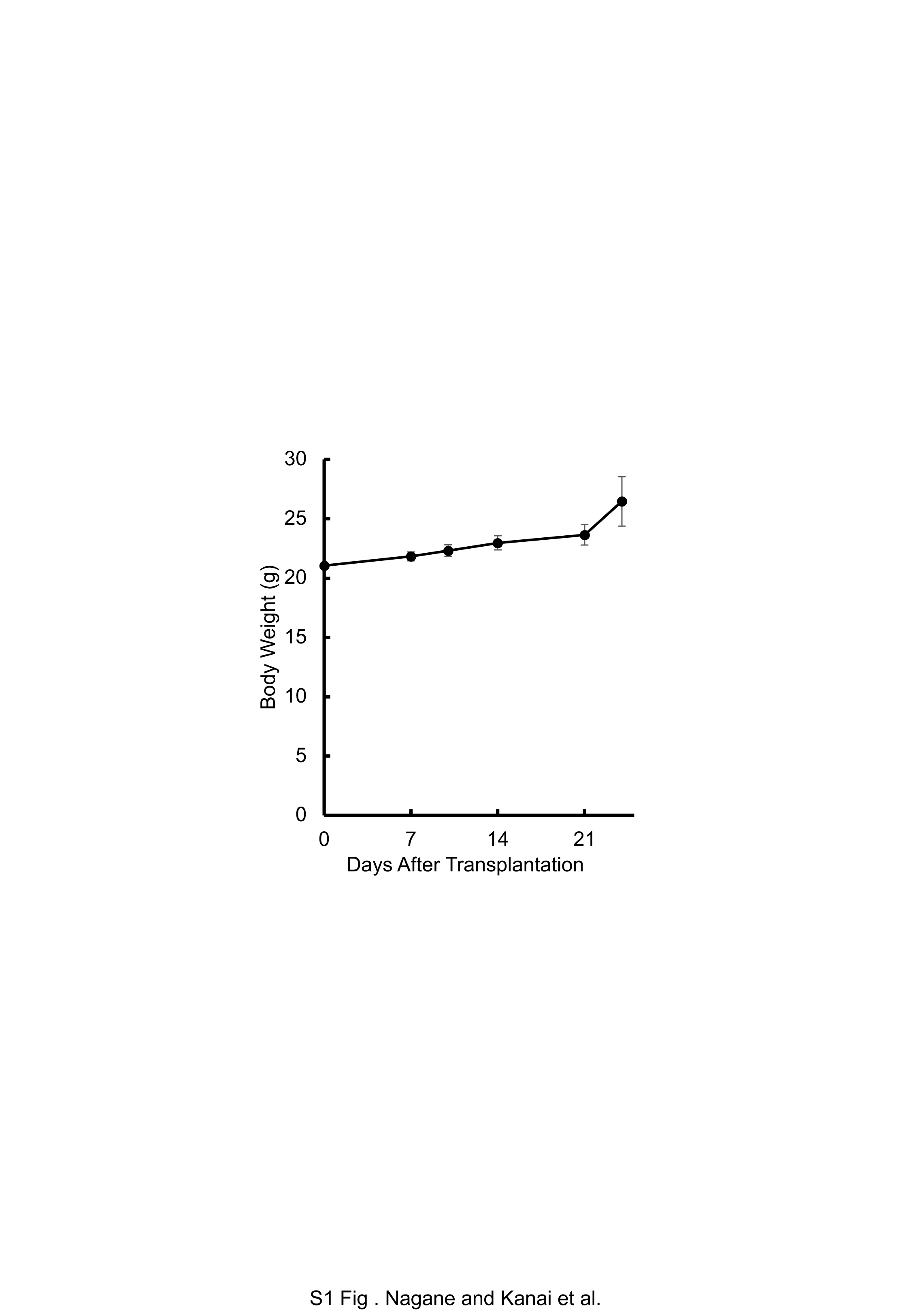

Supplement: S1 Fig — Body weight were measured after tumor transplantation. (TIF) [file pone.0195151.s003.tif]

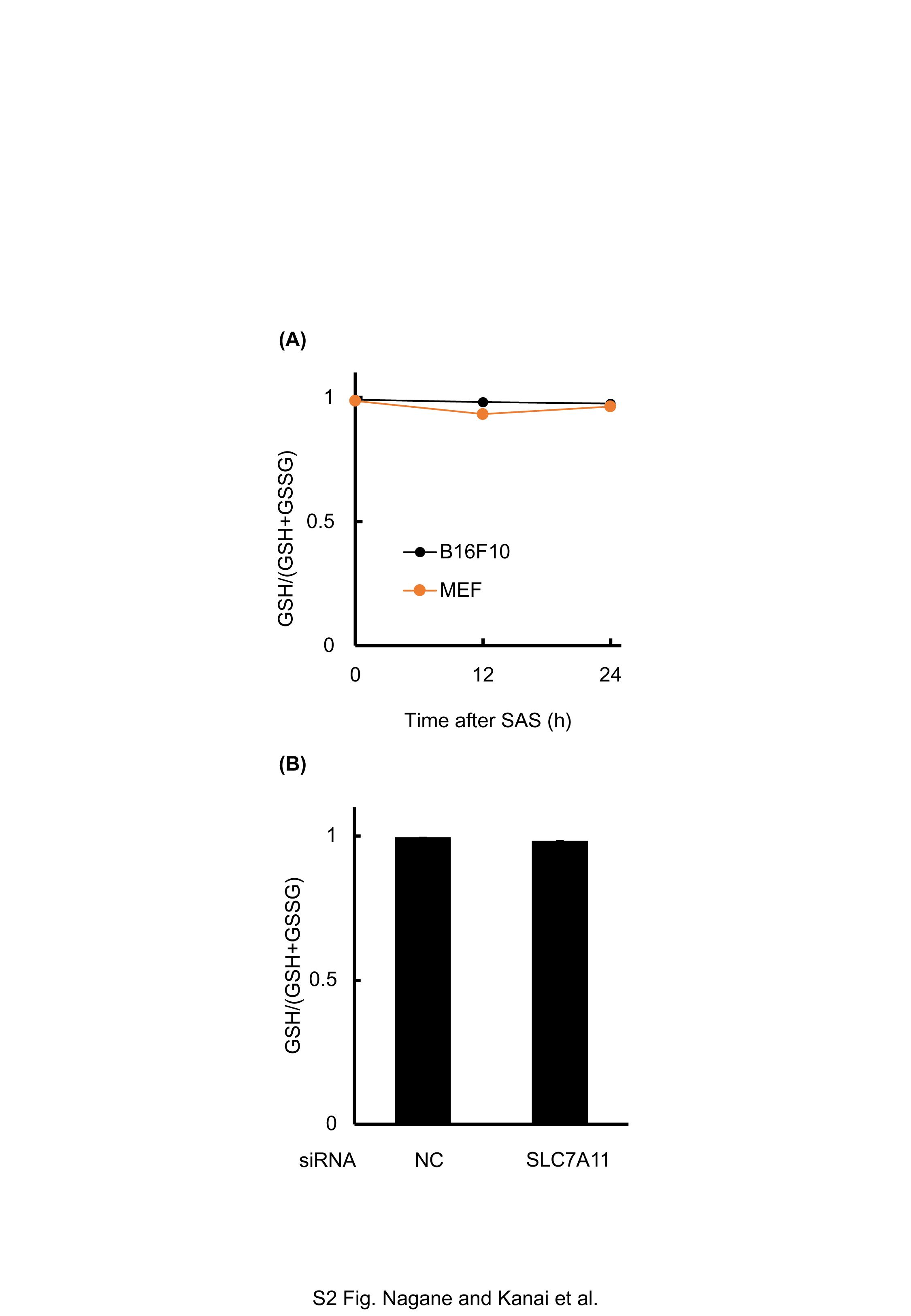

Supplement: S2 Fig — GSH/(GSH+GSSG) were analyzed after xCT inhibition. (A) Cells were treated by SAS (200 μM, 24 h) and analyzed GSH+GSSG concentration and GSSG concentration. (B) Cells were treated by siSLC7A11 (30 nM, 72 h) and analyzed GSH and GSSG ratio. (TIF) [file pone.0195151.s004.tif]

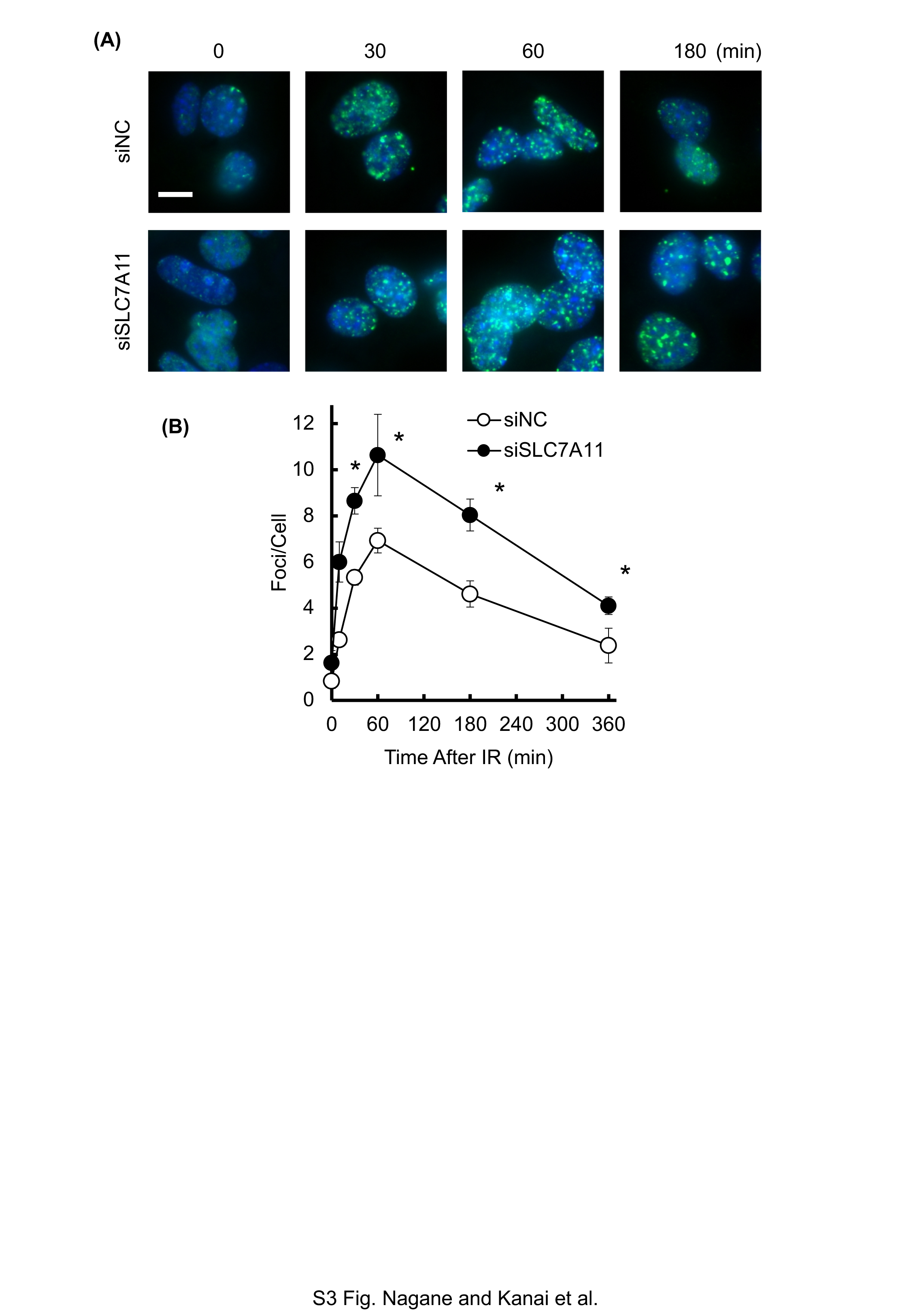

Supplement: S3 Fig — Cells were treated by siSLC7A11 (30 nM, 72 h) and irradiated at dose of 1 Gy. Then, cells were stained by phospho-H2AX. (A) representative image of phospho-H2AX after X-irradiation. White bar = 10 μM. (B) quantitative results of H2AX foci formation. Error Bar = S.D., p* < 0.05. (TIF) [file pone.0195151.s005.tif]

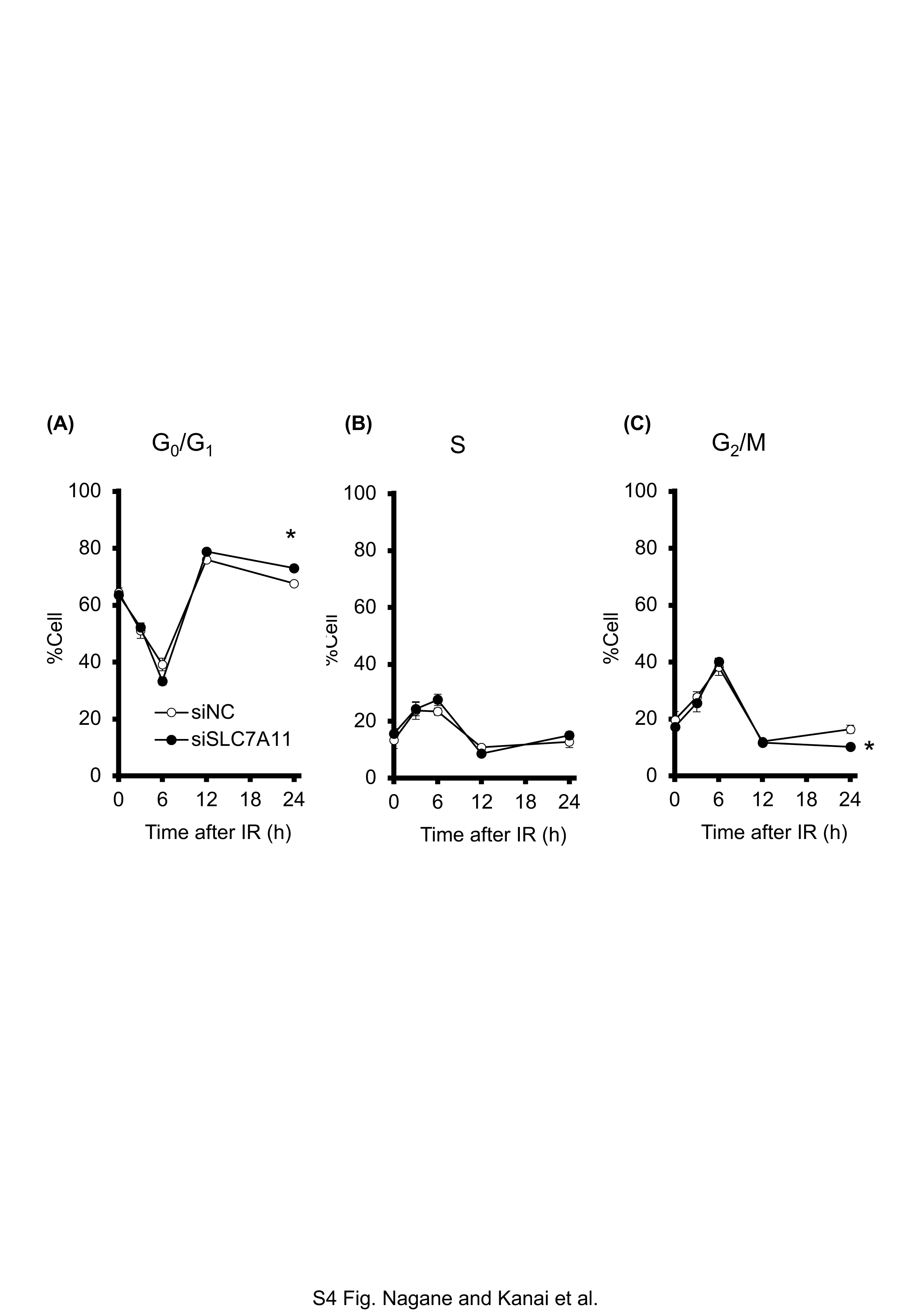

Supplement: S4 Fig — Cells were treated by siSLC7A11 (30 nM, 72 h) and irradiated at dose of 1 Gy. Then, cells were fixed at indicated time. Populations of (A) G0/G1-phase, (B) S-phase, and (C) G2/M-phase were analyzed by propidium iodide staining and flow cytometry. Error bars = SD, *p<0.05. (TIF) [file pone.0195151.s006.tif]

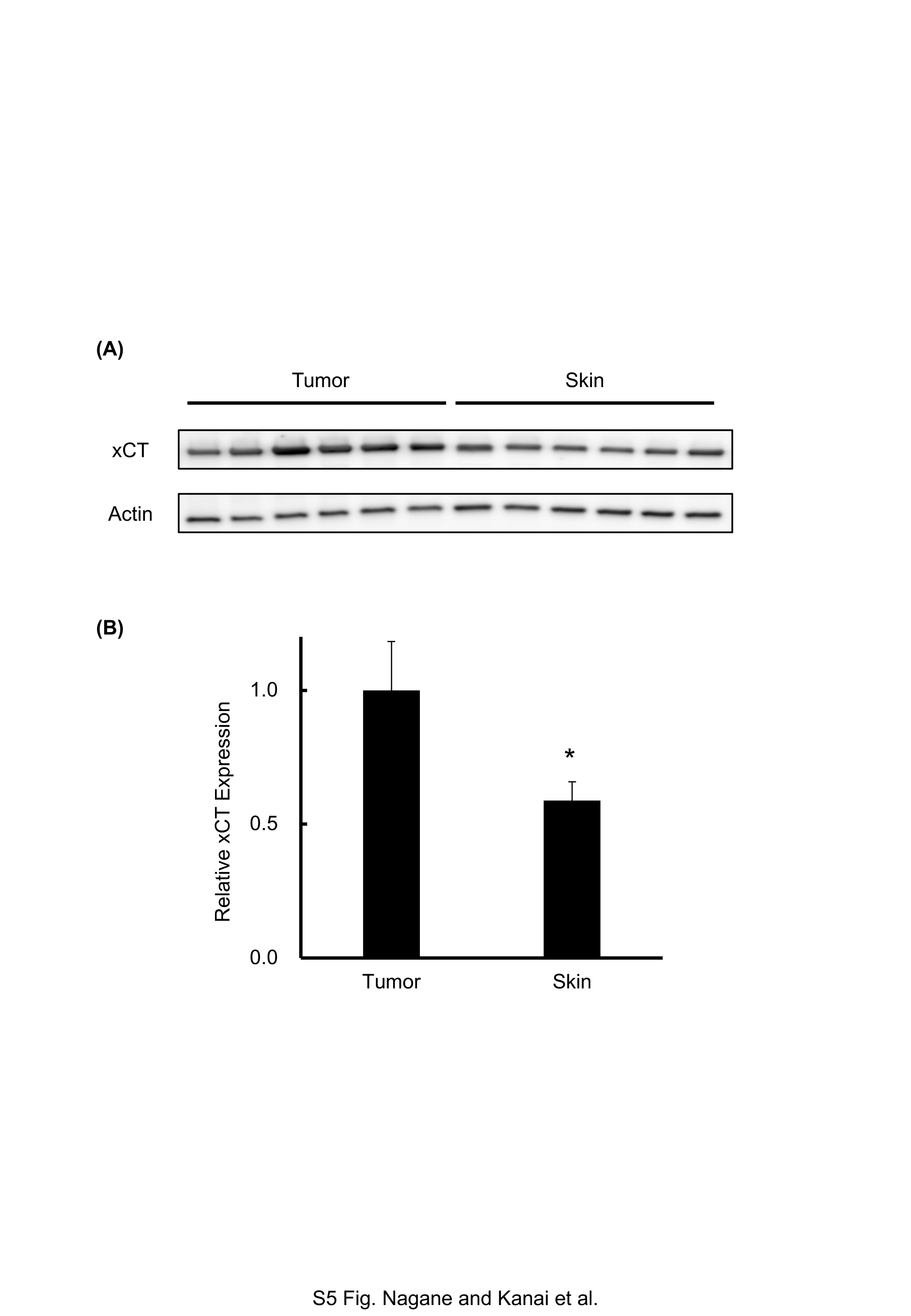

Supplement: S5 Fig — B16F10 tumors were excised and lysed in RIPA buffer. Expression of xCT were measured by western blot. (A) image of western blot for 6 tumors and 6 skin tissues. (B) Quantitative analysis of band intensity of xCT. Bar = S.D., p* < 0.05. (TIF) [file pone.0195151.s007.tif]

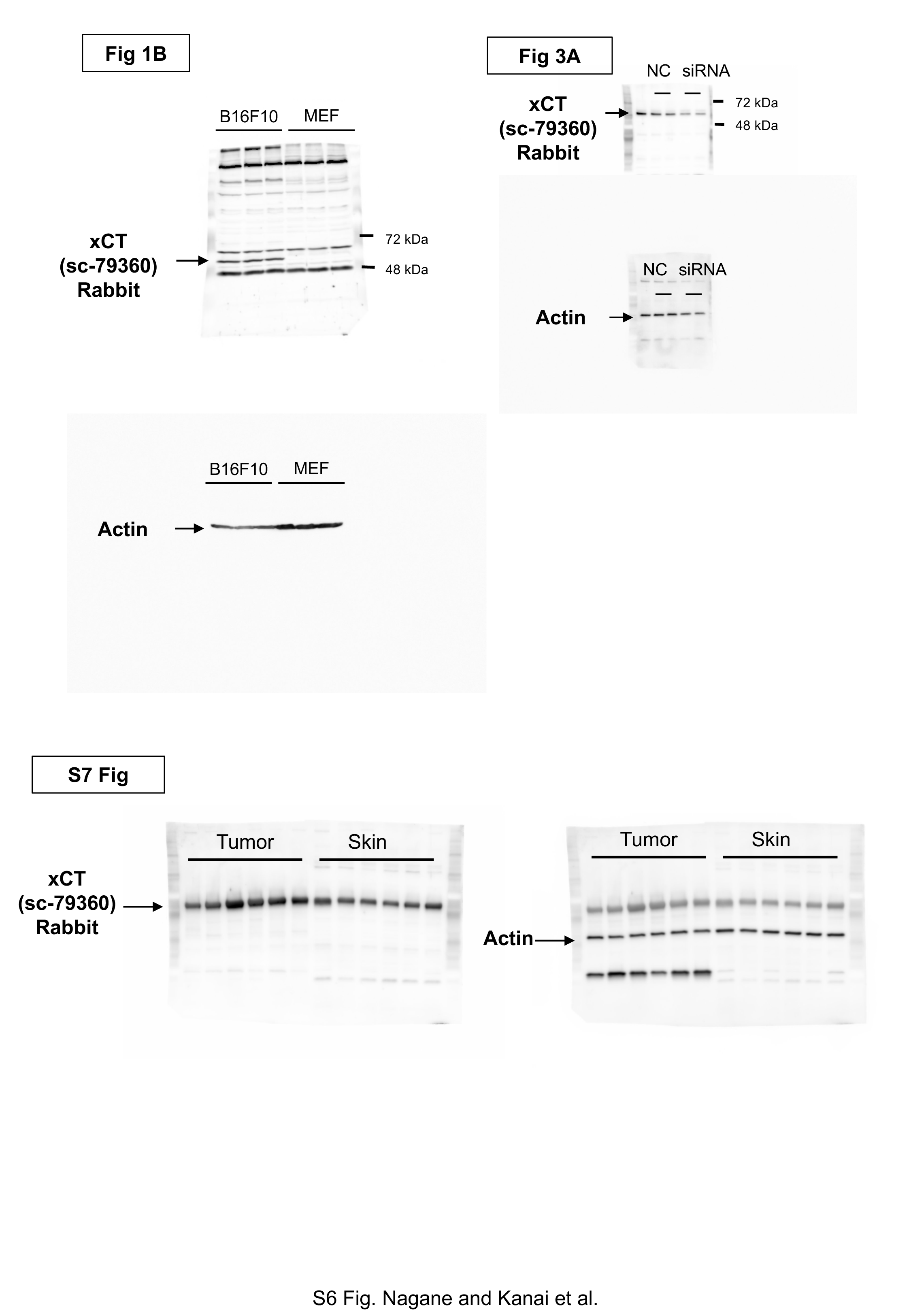

Supplement: S6 Fig — (TIF) [file pone.0195151.s008.tif]
